# Effects of rations containing formaldehyde-protected soybean meal on meat production in Kacang goats

**DOI:** 10.14202/vetworld.2019.890-895

**Published:** 2019-06-25

**Authors:** Retno Adiwinarti, I. Gede Suparta Budisatria, K. Kustantinah, R. Rusman, Edwin Indarto

**Affiliations:** 1 Department of Animal Science, Faculty of Animal and Agricultural Sciences, Diponegoro University, Semarang, Indonesia; 2 Department of Animal Production, Faculty of Animal Science, Gadjah Mada University, Yogyakarta, Indonesia; 3 Department of Animal Nutrition and Feeds Science, Faculty of Animal Science, Gadjah Mada University, Yogyakarta, Indonesia; 4 Department of Animal Product Technology, Faculty of Animal Science, Gadjah Mada University, Yogyakarta, Indonesia

**Keywords:** carcass, chevon quality, daily gain, Kacang goat, soybean meal

## Abstract

**Aim:**

This study aimed to investigate effects of rations containing formaldehyde-protected soybean meal on meat production in Kacang goats.

**Materials and Methods:**

Fourteen yearling Kacang bucks, weighing 15.8-19.8 kg, were arranged in a completely randomized design. The treatments included a control (PSBM0): 100% untreated SBM; PSBM50: 50% untreated SBM + 50% formaldehyde-protected SBM; and PSBM100: 100% formaldehyde-protected SBM.

**Results:**

The goats disliked the protected SBM. Therefore, differences in their intakes were reflected in their average daily gain (ADG). The ADG and slaughtered weight of the control group were the highest, while those of the PSBM100 and PSBM50 groups were similar. The carcass weights and meat production of the control group were higher than those of the PSBM50 group, but the retained protein to the meat conversion ratio of the PSBM50 group was lower than that of the control. The carcass percentages were similar between the treatments.

**Conclusion:**

The retained protein to meat conversion ratio of Kacang goats fed with 50% formaldehyde-protected SBM showed the lowest value, indicating that these rations efficiently produced meat in the carcass.

## Introduction

Genetic structure and environmental factors both have effects on productivity [1]. Kacang goats are one of the indigenous goat species in Indonesia, raised traditionally in rural areas. The productivity of some Kacang goats is low because the goats are poorly fed and graze on natural grass. The performance of Kacang goats has been improved using soybean meal (SBM) and fish meal in rations [2]. Adiwinarti *et al*. [2] reported that the dry matter intake of rations containing SBM was higher than those of rations containing fish meal. However, SBM is highly degradable in the rumen [3]. The dry matter digestibility of a rice straw diet supplemented with SBM for goats was 59.5% [4]. In addition, the degradability of SBM, using an *in situ* technique, in Cashmere goats after incubation for 12 and 24 h was approximately 64.75 and 80.57% of the dry matter, respectively [5].

Many efforts to protect feed from degradation in the rumen have been attempted [6]. Formaldehyde is a chemical used in animal nutrition, is environmentally safe, [7] and can lower protein degradation in the rumen [8]. Mahima *et al*. [8] reported that the use of 1.5% formaldehyde protected mustard oil cake and increased the *in vitro* digestibility of indigestible protein in wheat straw. Formaldehyde can also decrease the *in vitro* degradability of SBM in the rumen. Suhartanto *et al*. [9] reported that the *in vitro* dry matter degradability of SBM was 89.9%, while that of 0.5% and 1% formaldehyde-protected SBM was 52.3% and 35.3%, respectively. Although formaldehyde-protected SBM has been applied in cattle [10-13] and sheep [14-17], its application in goats is still limited. However, there has been researching on using formaldehyde protection in goats for sesame cake instead of SBM [18].

Beigh *et al*. [19] studied total mixed rations (TMR) to improve the intake and nutrient utilization of ruminants. The TMR comprised blended concentrate, roughage [19], a protein source, minerals, vitamins to form balance, and economical rations [19-21]. Feed with low palatability can be mixed in TMR to increase the intake [21]. Feed efficiency increased by approximately 4% when using TMR compared to concentrate and roughage given separately [20].

Therefore, this research studied the effects of SBM protected by 1% formaldehyde in TMR on the growth, carcass production, and chevon quality of Kacang goats. To increase the productivity of goats, the optimum ratio of protected SBM as a function of the physical and chemical properties was explored.

## Materials and Methods

### Ethical approval

The procedures concerning the use of animals in this experiment were approved by the Animal Ethics Committee of the Faculty of Animal and Agricultural Sciences, Diponegoro University, Semarang, Indonesia.

### Materials

Fifteen Kacang bucks, a year of age (indicated by having a total of two permanent incisors), were arranged in a completely randomized design. However, one of the goats in the PSBM100 group died during the research; therefore, only 14 goats were used in this research. Their body weights ranged from 15.8 to 19.8 kg, with an average of 17.6±1.2 kg. The rations consisted of 30% *Pennisetum purpureum*, 30% *Gliricidia* leaves, 19.2% cassava waste products, 13.8% wheat bran, 7% SBM, and 1% mineral mix. In addition, it contained 14-15% crude protein and 56-60% total digestible nutrients.

### Methods

A completely randomized design was used in this experiment. The treatments included a control or PSBM0 group: 100% untreated SBM; PSBM50 group: 50% untreated SBM + 50% formaldehyde-protected SBM; and PSBM100 group: 100% formaldehyde-protected SBM.

The SBM was protected with 1% formaldehyde. Formalin, containing 37% formaldehyde diluted 4 times with water, was sprayed on the SBM, and then the treated SBM was fermented overnight. Afterward, the SBM was aerated for 1 day and sun-dried for 2 days. The goats were weighed weekly, over 94 days, and the average daily gain (ADG) was calculated using linear regression [2].

The retained protein was calculated from the protein intake minus the fecal and urinary protein. Between the 8^th^ and 10^th^ weeks of the experiment, daily feces and urine were collected, weighed, and sampled. The procedures used for the fecal and urine sampling were based on those of Darlis *et al*. [4] and Elamin *et al*. [22], respectively.

Goats were weighed and slaughtered after a 12 h fast, with free access to drinking water. The goats were slaughtered, according to Pratiwi *et al*. [23], but carcasses in this research included the kidney and surrounding fat. Carcasses were weighed and deboned to evaluate the total bone, fat, meat, meat to bone ratio, and retained protein to meat conversion ratio. The retained protein to meat conversion ratio was calculated as the retained protein divided by the meat product.

Biceps femoris muscles were used for the physical and chemical quality assays, which were based on the methods of Mirdhayati *et al*. [24]. The physical qualities of chevon included the pH, water-holding capacity (WHC), cooking loss, and tenderness obtained from Warner-Bratzler shear force value, which were observed based on the methods of Shirima *et al*. [25] and Moawad *et al*. [26]. The chemical qualities of chevon included the water, fat, protein, and collagen content, which were determined using near-infrared spectroscopy [27]. Data were analyzed as a completely randomized design using one-way ANOVA [28].

## Results

### The ADG, carcass, and meat production

**Figure-1:**
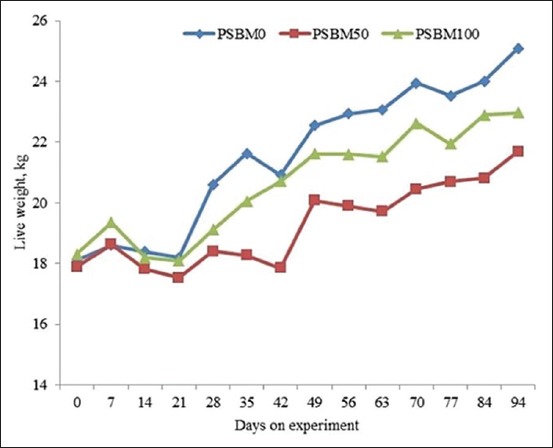
Body weight of Kacang goats receiving formaldehyde-protected soybean meal 0% (PSBM0), 50% (PSBM50), and 100% (PSBM100).

The dry matter intake (DMI) of the PSBM50 group was lower (p<0.01) than that of the control (PSBM0) group, however, the DMI of the PSBM50 and PSBM0 groups was not significantly different (p>0.05) from that of the PSBM100 group (Table-1). Differences in their intakes were reflected in their live weight (Figure-1), ADG, slaughter weight, carcass weight, and meat weight (Tables-1 and 2). The live weight, ADG, slaughtered weight, carcass weight, and meat weight of the PSBM0 group were higher than those of the PSM50 and PSM100 groups (Figure-1), while those of the PSBM50 and PSBM100 groups were about same (Tables-1 and 2).

**Table-1:** The DMI, ADG, slaughter weight, carcass weight, and carcass percentage.

Parameters	PSBM0 (control)^1^	PSBM50^2^	PSBM100^3^	p-value
DMI, g	701.3±81.6^A^	541.1±41.6^B^	606.9±65.9^AB^	0.008
ADG, g	78.9±10.3^A^	41.5±15.2^B^	56.5±5.0^B^	0.001
Slaughter weight, kg	25.1±1.3^A^	21.7±1.1^B^	23.0±0.6^B^	0.001
Carcass weight, kg	11.6±1.0^A^	9.5±0.8^B^	10.1±0.4^AB^	0.006
Carcass percent, %	46.2±3.6	43.8±2.1	43.8±2.1	0.337

DMI=Dry matter intake; ADG=Average daily gain.

A,B within a row, means without a common uppercase superscript differ (p<0.01).

1 PSBM0 (control) means 100% untreated SBM.

2 PSBM50 means 50% untreated SBM+50% formaldehyde-protected SBM.

3 PSBM100 means 100% formaldehyde-protected SBM. SBM=Soybean meal

**Table-2:** Carcass components and meat to bone ratio.

Parameters	PSBM0 (control)^1^	PSBM50^2^	PSBM100^3^	p-value
Meat, kg	8.1±0.9^a^	6.5±0.6^b^	7.2±0.1^a,b^	0.013
Meat, %	71.1±3.1	69.6±1.3	73.1±1.4	0.086
Fat, kg	1.1±0.2	0.9±0.1	0.7±0.3	0.099
Fat, %	9.5±2.5	9.2±0.7	7.4±2.6	0.320
Bone, kg	2.2±0.2	2.0±0.1	1.9±0.2	0.112
Bone,%	19.4±1.6	21.2±1.5	19.5±1.7	0.182
Meat+fat to bone ratio	4.2±0.4	3.7±0.3	4.2±0.4	0.187
Meat to bone ratio	3.7±0.4	3.3±0.3	3.8±0.3	0.124

a,b Within a row, means without a common uppercase superscript differ (p<0.05).

1 PSBM0 (control) means 100% untreated SBM.

2 PSBM50 means 50% untreated SBM+50% formaldehyde-protected SBM.

3 PSBM100 means 100% formaldehyde-protected SBM. SBM=Soybean meal

The carcass and meat weight of the PSBM50 group were lower than those of the PSBM0 group. Surprisingly, although the meat weight of the PSBM50 group was lower than that of the PSBM0 group, the retained protein to the meat conversion ratio of the PSBM50 group was the lowest (Table-3). The lower the value of the retained protein to meat conversion ratio, the better the ration, because this indicates that the amount of retained protein required to convert a gram of meat was lower.

**Table-3:** Retained protein to meat conversion ratio.

Parameters	PSBM0 (control)^1^	PSBM50^2^	PSBM100^3^	p-value
Retained protein, g	59.5±19.3^A^	24.1±2.8^B^	42.4±2.4^AB^	0.002
Meat, g	8078.6±978.9^a^	6472.5±615.7^b^	7226.7±141.0^ab^	0.013
Retained protein to meat conversion ratio	0.007±0.002^A^	0.004±0.001^Bb^	0.006±0.000^Aa^	0.002

A,B Within a row, means without a common uppercase superscript differ (p<0.01).

a,b Within a row, means without a common uppercase superscript differ (p<0.05).

1 PSBM0 (control) means 100% untreated SBM.

2 PSBM50 means 50% untreated SBM+50% formaldehyde-protected SBM.

3 PSBM100 means 100% formaldehyde-protected SBM. SBM=Soybean meal

The carcass and meat weight of the PSBM50 and PSBM0 groups were not significantly different (p>0.05) from the PSBM100 group (Tables-1 and 2). In fact, the carcass and meat percentage were similar between the treatments (Tables-1 and 2). The averages of the carcass and meat percentages were 44.6 and 71.2%, respectively. The fat, bone, meat + fat to bone ratio, and meat to bone ratio were also similar between the treatments (Table-2), with average values of 0.9 kg (8.7%), 2.0 kg (20.0%), 4.0, and 3.6, respectively.

### The chevon quality

The physical qualities of the chevon were almost the same between the treatments, with average values of pH 6.0, WHC 39.8%, cooking loss 37.2%, and tenderness 6.8 kg/cm^2^ (Table-4). The chemical qualities of the chevon were also the same between the treatments, with average values of moisture 72.8%, protein 21.6%, fat 2.6%, and collagen content 1.9% (Table-4).

**Table-4:** Physical and chemical quality of the chevon.

Parameters	PSBM0 (control)^1^	PSBM50^2^	PSBM100^3^	p-value
Physical quality				
pH	6.0±0.1	6.0±0.1	6.0±0.1	0.890
WHC, %	39.1±1.9	40.2±2.0	40.4±3.9	0.740
Cooking loss, %	37.1±2.5	38.0±2.3	36.3±3.1	0.620
Tenderness, kg/cm^2^	6.9±0.1	6.8±0.3	6.8±0.3	0.820
Chemical quality				
Moisture, %	72.3±0.8	72.9±1.1	73.2±0.9	0.383
Protein, %	22.2±0.9	21.5±0.7	21.0±0.8	0.150
Fat, %	2.8±0.4	2.5±0.6	2.3±0.5	0.420
Collagen, %	1.9±0.2	2.0±0.2	2.0±0.1	0.653

WHC=Water-holding capacity.

1 PSBM0 (control) means 100% untreated SBM.

2 PSBM50 means 50% untreated SBM+50% formaldehyde-protected SBM.

3 PSBM100 means 100% formaldehyde-protected SBM. SBM=Soybean meal

## Discussion

### The ADG, carcass, and meat production

The ADG of the goats was influenced by the feed intake. Goats that consumed more feed showed a higher body weight gain. The lower intake of goats fed with rations containing formaldehyde-protected SBM (PSBM50 and PSBM100) indicates that Kacang goats did not like the palatability of the rations because goats are selective feeders, as stated by Rahman *et al*. [29]. Although the DMI of the goats fed formaldehyde-protected SBM (541.1 and 606.9 g for PSBM50 and PSBM100, respectively) in this research were lower than those of the goats in Rahman *et al*. [29], their ADG was higher (41.5 and 56.5 g for PSBM50 and PSBM100, respectively, vs. 30.8-43.5 g for Rahman *et al*. research). The ADG of the goats fed PSBM0 (78.9 g) was higher than that of the Kacang goats reported by Restitrisnani *et al*. [30] (23.5-69.4 g). However, Rahman *et al*. [31] reported that crossbred Boer goats fed with soy waste products and *P. purpureum* had a weight gain of 80.2 g/d.

The range of carcass weights of Kacang goats in this research (9.5-11.6 kg) was relatively similar to that of Kacang goats in Gafar *et al*. [32] (10.7-12.2 kg). However, Kacang goat carcasses were heavier than Indian local goat carcasses, as Solanki *et al*. [33] reported that Indian local goats had carcass weights of 6.1-7.2 kg at 6-7 months and slaughter weights of 13.2-15.0 kg. In contrast, Hwangbo *et al*. [34] reported that Korean Black goats had carcass weights of 16.3-17.0 kg at 6 months and slaughter weights of 31.0-31.7 kg. Based on these studies, it can be concluded that carcass weights are influenced by the slaughter weight, age at slaughter, and breed. In addition, Yusuf *et al*. [35] reported that a heavier carcass was due to higher feed intake and better weight gain. Kacang goats are a local Indonesian goat that has a small body size (Kacang means that the goat is as small as a peanut); therefore, the carcass weight is also low. However, because they are prolific and adaptable to poor feeding management, Kacang goats are widely reared by farmers in villages.

The carcass percentages were similar between the treatments. This indicates that the carcass percentage was influenced by the carcass and slaughter weights. The carcass percentages of Kacang goats in this study (43.8-46.2%) were lower than those of Kacang goats reported by Gafar *et al*. [32] (53.3-56.8%), or other breeds reported by Johnson *et al*. [36] (49.4-49.9%) and Hwangbo *et al*. [34] (51.6-54.4%). However, they were relatively similar to those reported by Hutama [37] (46.7%), Solanki *et al*. [33] (46.0-48.2%), Singh *et al*. [38] (44.7-47.6%), Yusuf *et al*. [35] (40.3-48.1%), and Akbaş and Saatci [39] (43.9-46.1%), while they were higher than those reported by Sumardianto *et al*. [40] (40.9%) and Adiwinarti *et al*. [41] (38.8%). The differences between the carcass percentages, which varied between 35% and 53%, were caused by the different feeds, feeding management systems, genotype, sex, and age [39]. However, Das and Rajkumar [42] reported that the dressing percentage was less influenced by the breed. In fact, the dressing percentage was mostly influenced by the slaughter weight [34,39].

Meat produced from the PSBM0 (control) group was more than that of the PSBM50 group (Table-2) due to the higher DMI, protein intake, and retained protein. However, the retained protein to the meat conversion ratio of the PSBM50 group was significantly lower (p<0.01) than that of the PSBM0 (control) group (Table-3). This indicated that the PSBM50 ration has the potential to increase meat production, but further research is needed to increase the DMI of the PSBM50 ration.

The meat percentages in this study (69.6-73.1%) were similar to those of the control goats in Yusuf *et al*. [35] (69.3%). However, they were lower than those of the goats fed *Andrographis paniculata* (83.1-84.1%) [35], and higher than those in the studies of Hwangbo *et al*. [34] (58.1-59.6%), Aktaş *et al*. [43] (53.7-57.9%), and Ayeb *et al*. [44] (54.7-61.7%). Ayeb *et al*. [44] stated that the diet did not directly influence the meat percentages, as the goats had similar slaughter weights.

In this study, the fat percentage (7.4-9.5%) was lower than that reported by Aktaş *et al*. [43] (8.8-12.4%) and Yusuf *et al*. [35] (9.5-21.8%). The fat percentage was influenced by the slaughter weight. Never [45] reported that heavier animals produce a higher proportion of fat and lower proportion of muscle and bone. In this study, the slaughter weight (21.7-25.1 kg) was lower than that reported by Aktaş *et al*. [43] (25.0-38.8 kg) and Yusuf *et al*. [35] (25.7-31.8 kg).

In this study, the bone percentage (1.9-2.2%) was higher than that in Hwangbo *et al*. [34] (18.1-18.9%) and Yusuf *et al*. [35] (8.4-12.0%). This difference is due to the different slaughter weights. Ayeb *et al*. [44] reported that the bone percentage was similar between the diet treatments as the goats have the same slaughter weight.

In this study, the meat to bone ratio of Kacang goats (3.3-3.8) was higher than that reported by Sumardianto *et al*. [40] (2.6) or Adiwinarti *et al*. [41] (2.2), but lower than that reported by Yusuf *et al*. [35] (6.2-9.9). Never [45] stated that the carcass composition was affected by the nutrient intake, feed composition, and nutrient requirements of the animal.

### The chevon quality

The physical quality of the carcass was not influenced by the diet [46]. The pH of chevon in this study was lower than that reported by Adiwinarti *et al*. [41] in traditionally managed Kacang goats (pH: 6.3). The high pH caused the high WHC, as Judge *et al*. [47] stated that the higher the pH (5.2-6.8), the more protein-bound water is present and the higher the WHC. The tenderness was similar between the treatments because the collagen contents were the same.

Hwangbo *et al*. [34] reported that the chemical compositions of chevon in Korean black goats were not different between the treatments (the level of protein content started from 14% to 20%). The water content was influenced by the fat content. The high water content in this study might be caused by the low-fat content of the chevon [24,34]. Mirdhayati *et al*. [24] reported that the water content of Kacang goats was 73.8-74.5% and the fat content was 0.4-0.5%, while Hwangbo *et al*. [34] reported that the moisture content of the Korean Black goat, fed TMR, was 74.3-74.8% and the fat content was 1.4-1.7%. The protein content (21.0-22.2%) was lower than that reported by Mirdhayati *et al*. [24] (23.2-23.5%), but relatively similar to that reported by Hwangbo *et al*. [34] (21.7-22.5%), and higher than that reported by Adiwinarti *et al*. [41] (19.6-19.7%).

## Conclusion

Goats fed PSBM0 had the best ADG, slaughter weight, carcass weight, and meat products due to the higher DMI. However, goats fed PSBM50 rations were the most efficient meat producers, which was indicated by the lowest retained protein to meat conversion ratio.

## Authors’ Contributions

RA conducted the experiment, acquisition of data, and drafting of the manuscript. IGSB advised in the design of the experiment, data analysis, and interpretation. KK developed the feeding concepts and supervised the experiment. RR advised on the analysis and interpretation of the post-mortem data. EI supervised nutrition analyses. All the authors accepted the final manuscript.
